# Virology Applications to the COVID-19 Pandemic

**DOI:** 10.3390/life15020247

**Published:** 2025-02-06

**Authors:** Evangelia Georgia Kostaki

**Affiliations:** Department of Hygiene, Epidemiology and Medical Statistics, Medical School, National and Kapodistrian University of Athens, 11527 Athens, Greece; ekostakh@med.uoa.gr

From the early identification of severe respiratory cases of unknown etiology in Wuhan, China, in late 2019, virology research has played an important role in understanding, management, and prevention of the COVID-19 pandemic. Firstly, virology applications were important in identifying SARS-CoV-2 as the causative agent, classifying the virus, and determining its closest viral relatives through whole-genome sequencing [1,2,3,4,5,6]. Secondly, virology research included critical applications such as (i) diagnostics development—the creation of molecular and antigen tests and the development of serological tests to detect antibodies [7,8,9], (ii) epidemiology and surveillance—the implementation of wastewater epidemiology for viral monitoring, genomic surveillance to identify and monitor variants of interest (VOIs) and variants of concern (VOCs), tracing the origin of SARS-CoV-2 transmission, investigating the role of social contacts in viral spread, and conducting molecular epidemiological studies to analyze transmission patterns [10,11,12,13,14]. Additionally, virology studies were applied in assessing public health measures, guiding the development of therapeutics and monoclonal antibodies, and enabling rapid vaccine development using different technologies. Furthermore, virology research has contributed to understanding viral pathogenesis and supports a One Health approach to studying zoonotic origins and preventing future pandemics [15,16]. These contributions highlight the essential role of virology in relation to the pandemic.

The Special Issue, “Virology Applications to the COVID-19 Pandemic”, published in *Life*, in the section “Epidemiology”, includes 14 original research and review articles on applications of virology to the COVID-19 pandemic. Four studies, authored by Chrysostomou and Aristokleous et al. [17], Chaintoutis and Chassalevris et al. [18], Lim et al. [19], and Chrysostomou et al. [20], focus on the development or the evaluation of novel laboratory methods for SARS-CoV-2 detection and characterization. Chrysostomou and Aristokleous et al. introduce a rapid method for the identification of various VOCs using a multiallelic spectral genotyping assay [17]. This method, based on real-time reverse transcription–PCR in combination with probes, offers several advantages versus next-generation sequencing, providing fast and accurate results. Similarly, Chaintoutis and Chassalevris et al. developed a one-step real-time RT-PCR assay to rapidly identify Alpha, Beta, Gamma, or Delta VOCs [18]. This assay employs four locked nucleic acid (LNA) modified TaqMan probes targeting signature mutations in the receptor-binding motif (RBM) of the spike protein’s receptor-binding domain (RBD). Validation with known SARS-CoV-2-positive and -negative samples demonstrated its accuracy in characterizing variants. Additionally, the assay can be adapted to detect a broader range of variants. The study by Lim et al. evaluated three automated nucleic acid extraction systems for SARS-CoV-2 detection: the MagNA Pure 96 DNA and Viral NA Small Volume kit (Roche, Basel, Switzerland) on the MagNA Pure 96 platform, the careGENETM Viral/Pathogen HiFi Nucleic Acid Isolation kit (WELLS BIO Inc., Seoul, Repulic of Korea) on KingFisher Flex (Thermo Fisher Scientific, Rocklin, CA, USA), and the SGRespiTM Pure kit (Seegene Inc., Seoul, Repulic of Korea) on Maelstrom 9600 (Taiwan Advanced Nanotech Inc., Taoyuan, Taiwan) [19]. Bland–Altman analysis showed high concordance among the platforms, with 95.2% concordance between MagNA Pure 96 and KingFisher Flex and 95.4% between MagNA Pure 96 and Maelstrom 9600, indicating statistically reliable results across all systems. Chrysostomou et al. presented a real-time RT-PCR detection assay designed to address the high genetic polymorphism of SARS-CoV-2 [20]. The assay employs mismatch-tolerant molecular beacons targeting the S, E, M, and N genes, enabling the detection of genetically diverse SARS-CoV-2 strains.

In a narrative review by Tofarides et al., the protective effect of vaccination against SARS-CoV-2 and long-COVID-19 was explored [21]. Current evidence indicates that vaccination reduces the risk of long-COVID-19, with the effectiveness influenced by factors such as the number of doses, the specific viral variant, the recency of vaccination, and, probably, age. Additionally, vaccination appears to lower the risk of neurocognitive–psychological disorders and cardiovascular complications. However, the potential role of the influenza vaccine in preventing long-COVID-19 remains unclear. Marot et al. assessed two surrogate neutralization assays to evaluate immune responses against the B.1, Alpha, Beta, and Omicron variants [22]. Their findings revealed the strongest neutralization responses in recovered COVID-19 patients who received a single vaccine dose. Naïve individuals who received two doses of an mRNA vaccine showed high neutralization titers against the B.1, Alpha, and Beta variants, though only 34.3% demonstrated activity against Omicron. On the other hand, non-infected individuals with an incomplete vaccination scheme exhibited weak and inconsistent neutralization activity across all variants. Terpos et al. investigated the kinetics of neutralizing antibodies (NAbs) six months after the second dose of the BNT162b2 mRNA vaccine [23]. At this timepoint, 2.59% of participants had NAb levels below 30%, 11.9% had NAb levels below 50%, and 58% had NAb levels above 75%. Older age was consistently associated with lower NAb levels across all timepoints. Population modeling predicted that 50% of individuals would have NAb levels below 73.8% at nine months and 64.6% at 12 months post-vaccination. The study highlights a sustained decline in humoral immunity six months after full vaccination, offering valuable insights for public health planning. In the study by Fischer et al., humoral and cellular immune responses against SARS-CoV-2 were evaluated in 41 COVID-19 convalescents with a high mean age of 54 ± 8.4 years [24]. Antibodies against SARS-CoV-2 were detectable in 95% of the participants up to 8 months post infection, though their levels showed a declining trend in most participants over time. A specific long-lasting cellular immune response was also observed in these individuals by stimulating immune cells with SARS-CoV-2-specific peptides, targeting the viral spike, membrane, and nucleocapsid proteins, and then measuring the release of interferon-γ (IFN-γ).

In their study, Balaska et al. present a mass screening program for the detection of SARS-CoV-2 by RT-PCR, performed on all professionals in a hospital, irrespective of symptoms [25]. The total number of samples tested was 43,726. The average positivity rate was 1.21% and was similar to the community positivity rate in Greece. Specifically, among the positive participants, 31% experienced no symptoms before receiving the positive result, 46.1% reported close contact with a patient or infected coworkers, and 32.8% reported close contact with infected family members. In periods of high COVID-19 incidence, the periodic testing of health care personnel can also be used to diagnose SARS-CoV-2 infections at the asymptomatic phase. Capozzi and Simone et al. report on an isolate from a 53-year-old woman who remained COVID-19-positive for approximately four months [26]. The viral isolate was assigned to lineage B.1.177.51 and was found to contain a novel set of mutations in the Spike protein (V143D, del144/145, and E484K). Seroneutralization assays revealed a significantly reduced response of this strain to both BNT162b2 Pfizer/BioNTech vaccine-induced antibodies (average reduction of 70%) and convalescent sera (average reduction of 19.04%) compared to VOC P.1. This study underscores the critical role of whole-genome sequencing (WGS) in tracking novel isolates from chronically infected individuals. Detsika et al. describe a cross-sectional study on the epidemiological, laboratory, and clinical characteristics of COVID-19 patients in relation to their immunogenetic profiles [27]. A statistically significant increase in HLA-DRB1*01 was detected in mild COVID-19 patients versus controls. The frequency of A*11, A*23, and DRB1*09 alleles was found to be higher, while the frequency of C*12 was lower, in hospitalized patients versus healthy controls, albeit with uncorrected statistical significance. Bonnet et al. explored whether the Alpha variant was associated with higher viral loads compared to the historical strain in saliva samples from patients with mild to moderate symptoms [28]. While a higher viral load was observed for the Alpha variant, no significant differences in viral load levels were detected between individuals infected with the Alpha variant and those with historical strains when accounting for the time interval between symptom onset and sampling.

In the study by Alshanbari et al., a machine learning approach (ML) was employed to investigate whether the ICU admissions of COVID-19 patients could be predicted [29]. The analysis used clinical and laboratory data from 100 patients diagnosed during May 2020 and January 2021. The study focused on the effectiveness of a weighted radial kernel support vector machine (SVM) coupled with recursive feature elimination (RFE). An initial assessment showed that the SVM with weighted radial kernels coupled with RFE outperformed other classification methods in discriminating between ICU and non-ICU admissions. Implementing RFE with weighted radial kernel SVM identified a significant set of variables that could predict and statistically distinguish ICU from non-ICU COVID-19 patients. These variables included patient weight, PCR Ct value, immune markers (CCL19, INF-β, BLC, INR), prothrombin time (PT), partial thromboplastin time (PTT), cardiac enzymes (CK-MB), blood parameters (HB, platelets, RBC), and biochemical markers such as urea, creatinine, and albumin levels. This study highlights the potential of weighted radial kernel SVM as a valuable ML tool to assist hospital decision-makers in optimizing resource allocation. Poonia et al. reported the use of an enhanced SEIR model designed to predict new cases of COVID-19 [30]. This model incorporates vaccination as an additional compartment, referred to as SEIRV, enabling predictions as to the severity of COVID-19 in vaccinated populations. The model was simulated under three distinct scenarios: (1) without social distancing, (2) with social distancing, and (3) with a combination of social distancing and vaccination. The results indicate an epidemic growth rate of about 0.06 per day, with the number of infected individuals doubling every 10.7 days. When social distancing measures were included, the model estimated a basic reproduction number (R_0_) of 1.3, highlighting the effectiveness of these interventions in reducing the spread of the virus.

The studies presented in this Special Issue highlight the importance of the application of virology to the COVID-19 pandemic, including diagnostic innovations, immune response characterization, epidemiological modeling, and resource optimization through machine learning.
